# Read, Write, Adapt: Challenges and Opportunities during Kinetoplastid Genome Replication

**DOI:** 10.1016/j.tig.2020.09.002

**Published:** 2021-01

**Authors:** Jeziel D. Damasceno, Catarina A. Marques, Jennifer Black, Emma Briggs, Richard McCulloch

**Affiliations:** 1 The Wellcome Centre for Integrative Parasitology, University of Glasgow, Institute of Infection, Immunity and Inflammation, Sir Graeme Davies Building, 120 University Place, Glasgow, G12 8TA, UK; 2 Institute for Immunology and Infection Research, School of Biological Sciences, University of Edinburgh, Edinburgh EH9 3FL, UK

**Keywords:** kinetoplastid, DNA replication, DNA repair, antigenic variation, adaptation

## Abstract

The genomes of all organisms are read throughout their growth and development, generating new copies during cell division and encoding the cellular activities dictated by the genome’s content. However, genomes are not invariant information stores but are purposefully altered in minor and major ways, adapting cellular behaviour and driving evolution. Kinetoplastids are eukaryotic microbes that display a wide range of such read–write genome activities, in many cases affecting critical aspects of their biology, such as host adaptation. Here we discuss the range of read–write genome changes found in two well-studied kinetoplastid parasites, *Trypanosoma brucei* and *Leishmania*, focusing on recent work that suggests such adaptive genome variation is linked to novel strategies the parasites use to replicate their unconventional genomes.

## Highlights

Polycistronic transcription dominates and shapes kinetoplastid genomes, inevitably leading to clashes with DNA replication. By harnessing the resultant DNA damage for adaptation, kinetoplastids have huge potential for dynamic read–write genome variation.
Major origins of DNA replication are confined to the boundaries of polycistronic transcription units in the *Trypanosoma brucei* and *Leishmania* genomes, putatively limiting DNA damage. Subtelomeres may lack this arrangement, generating read–write hotspots.
In *T. brucei*, early replication of the highly transcribed subtelomeric variant surface glycoprotein (VSG) expression site may ensure replication-transcription clashes within this site to trigger DNA recombination, an event critical for antigenic variation.
*Leishmania* genomes show extensive aneuploidy and copy number variation. Notably, DNA replication requires recombination factors and relies on post-S phase replication of subtelomeres.
Evolution of compartmentalised DNA replication programmes underpin important aspects of genome biology in kinetoplastids, illustrating the consolidation of genome maintenance strategies to promote genome plasticity.

## Read–Write Genome Activity in Kinetoplastids

The genome contains all information necessary to direct every aspect of cell function [1]. Consequently, genome content must be maintained and transmitted through cell division, which requires **DNA replication** (see Glossary) to generate new genome copies and **DNA repair** to tackle genome damage. Impairing either process can cause genome instability and lead to reduced fecundity, disease, or lethality [2]. Nevertheless, genome composition changes over time, either incrementally or suddenly. Indeed, genome sequence and organisation are frequently deliberately changed – by a range of biological processes – to effect phenotypic variation in widespread aspects of organism function. Such directed changes make clear the genome is not a sacrosanct information store but, instead, a read–write resource for organism adaptation [3]. Examples of adaptive genome alterations that are critical for organism growth, development, and survival include mating type switching in fungi [4], immune gene maturation in vertebrates [5], **ploidy** changes [6] and genome fragmentation [7] during the life cycles of apicomplexan and ciliate microbes, and surface antigen gene switching in many pathogens to allow host immune evasion [8].

Eukaryotic single-celled microbes of the class Kinetoplastea share a concatenated and fragmented mitochondrial genome [9]. Several kinetoplastids are major human and animal parasites and are, understandably, the focus of most research, though relatives that infect only insects or are free-living occupy diverse habitats across Earth [10,11]. Kinetoplastids undergo a rich variety of read-write genome alterations that occur across their cell and life cycles. African trypanosomes, such as *Trypanosoma brucei*, evade elimination by the mammalian host adaptive immune response through **antigenic variation**. This locus-targeted read–write adaptive process involves recombination of variant surface glycoprotein (*VSG*) genes into telomeric *VSG* expression loci to alter the expression of the variant surface glycoprotein (*VSG*) coat that covers the parasite’s surface [12]. Antigenic variation in *T. brucei* relies on a huge library of *VSG* genes and pseudogenes that are dispersed across subtelomeres [13,14], which account for perhaps 50% of the genome, contain other repetitive genes and elements, and are notably variable in sequence content between chromosome homologues [15] and isolates [16]. These pronounced levels of read–write activity across the VSG-rich subtelomeres appear to be absent in the *T. brucei* genome core. By contrast, *Leishmania* displays genome-wide adaptive variation. Single- or multiple-gene copy number changes [17,18], including by formation of circular or linear **episomes**, arise via repeat sequences spread across the genome [19]. In parallel, fluctuating levels of whole chromosome **aneuploidy** are seen [20], affecting different chromosomes in different cells within a population [21]. Both forms of variation alter gene expression during the *Leishmania* life cycle and in response to drug pressure [22,23]. Several recent reviews have discussed the cellular machineries that influence read–write genome variation in *T. brucei* and *Leishmania* [12,18,24., 25., 26., 27.]. Genome maintenance and transmission in these parasites is increasingly analysed using next generation sequencing approaches and so, in this review, we discuss how *T. brucei* and *Leishmania* balance genome preservation and variation; in particular, asking if read–write genome activities are influenced or directed by challenges and novelties in how kinetoplastids replicate their genomes.

## The Unusual Genome Biology of Kinetoplastids

**Figure 1:**
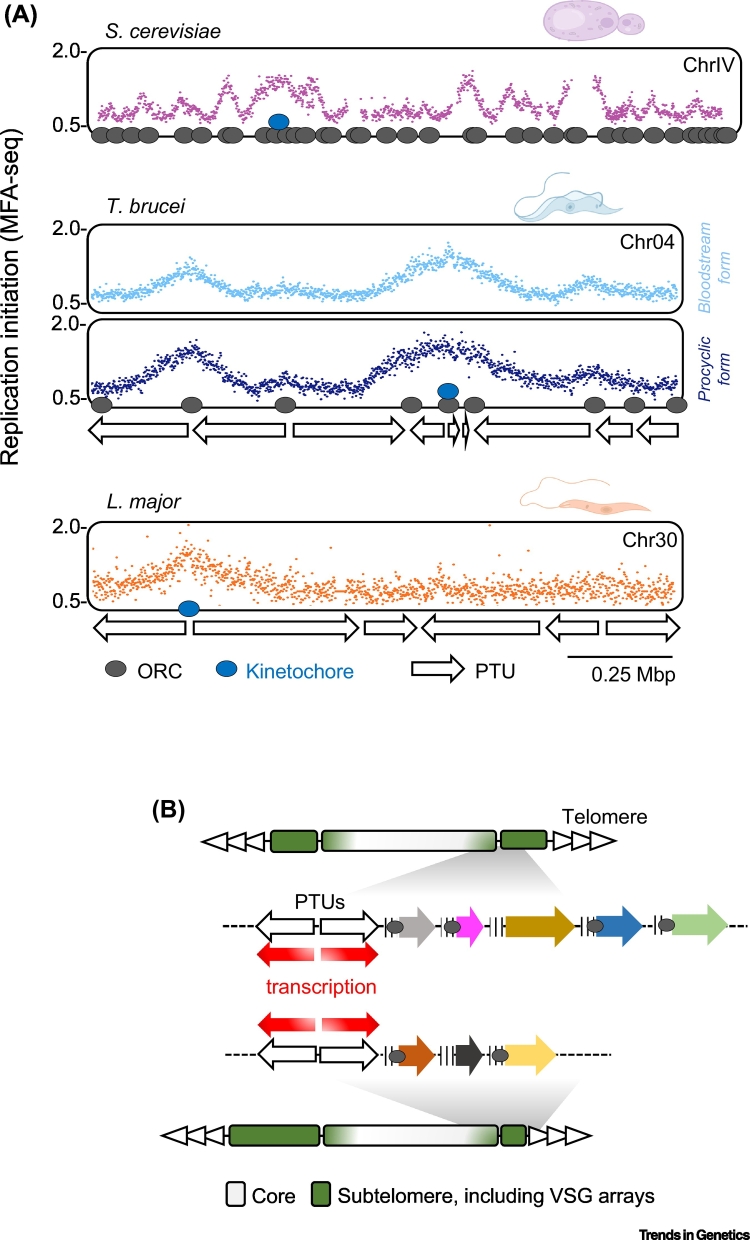
Key Figure. DNA Replication Programmes of *Trypanosoma brucei* and *Leishmania* (A) Examples of MFA-seq mapping of DNA replication are shown for single, similarly sized chromosomes in *Saccharomyces cerevisiae* (top), *T. brucei* (middle), and *Leishmania major* (bottom); for *T. brucei* MFA-seq is shown in two life cycle stages. Data for the three species can be found in, respectively, Muller *et al*. [112], Devlin *et al.* [46], and Marques *et al*. [76]. Peaks across the MFA-seq profiles represent regions where reads are enriched in replicating cells relative to non-replicating cells, and therefore denote sites where DNA replication initiates and proceeds bidirectionally. In both *T. brucei* and *L. major*, genes are arranged in multigene PTUs (represented by white arrows); genes are not depicted for *S. cerevisiae*. In *S. cerevisiae* approximate positions of ORC binding are denoted by grey circles, while ORC1/CDC6 binding is similarly shown at the ends of the PTUs in *T. brucei*. In all species binding of the kinetochore (blue circle) at a single centromere is indicated. (B) A schematic diagram comparing two chromosome homologues in *T. brucei*, illustrating the highly transcribed core with syntenic PTUs and the surrounding, transcriptionally silent subtelomeres, which show variation in size between the homologues and display little synteny between VSG genes and pseudogenes (coloured arrows). ORC1/CDC6 (grey circles) is thought to bind around the VSGs found in subtelomeres but its precise binding locations are unclear (binding at ends of core PTUs is not shown). Abbreviations: MFA-seq, marker frequency analysis coupled with deep sequencing; ORC, origin recognition complex; PTU, polycistronic transcription unit; VSG, variant surface glycoprotein.

Kinetoplastids are remarkable for using several novel strategies in core cell biology [28], with perhaps the most famous being the complex structure and mechanisms for maintenance and expression of their mitochondrial genome (Box 1). However, novelty relative to other eukaryotes is also found in the organisation and transmission of the kinetoplastid nuclear genome. Unlike in most eukaryotes, where each protein is normally encoded from a single transcription unit with its own promoter and terminator, virtually every gene in kinetoplastids – including the majority encoded by RNA polymerase (Pol) II – is transcribed as part of a polycistronic transcription unit (PTU), which can cover hundreds of genes (Figure 1A, Key Figure). RNA Pol II transcription appears constitutive and PTUs do not possess clear promoter sequences, but instead use dispersed, bidirectional start sites marked by accumulation of histone variants, ordered nucleosomes [29] and RNA–DNA hybrids (**R-loops**) acted up by **RNase H2** [30,31]. Transcription termination at the ends of PTUs is associated with a novel modified base, termed J, which recruits a recently described protein complex [32]. Remarkably, some protein-coding genes in *T. brucei*, including *VSGs* (see later), are expressed from multigene units transcribed by RNA Pol I, where the promoters share some homology with those at rRNA gene clusters [33]. Across kinetoplastids, lineage-specific genes have expanded into families [34]. In *T. brucei*, the thousands of *VSG* genes and pseudogenes that have arisen are accommodated by genome compartmentalisation. Each of the 11 megabase-sized diploid chromosomes contain mainly transcriptionally silent *VSG*-containing subtelomeres, which are organised as compact compartments that appear to be spatially distinct in the nucleus from the highly transcribed, PTU-containing chromosome cores (Figure 1B) [15]. Moreover, silent *VSG*s are also found on hundreds of intermediate and minichromosomes, which are structurally simpler than the megabase chromosomes and are segregated during mitosis by a distinct mechanism [35].

Box 1 **Divergent Biology of Kinetoplastids**

A schematic structure of a *Leishmania* promastigote cell is shown (Figure I), highlighting the range of cellular activities that differ from what has been characterised in most eukaryotic cells. Two key complexes in nuclear genome transmission – the origin recognition complex (Box 2) and the kinetochore – lack clear homology with other eukaryotes. The insert diagram depicts RNA Pol II-directed multigenic transcription and subsequent reactions to generate mature mRNAs: *trans*-splicing excises each coding sequence by addition of a 39 nucleotide splice leader, adding a 5′ cap, and leading to polyadenylation of the upstream RNA molecule.

**Figure I:**
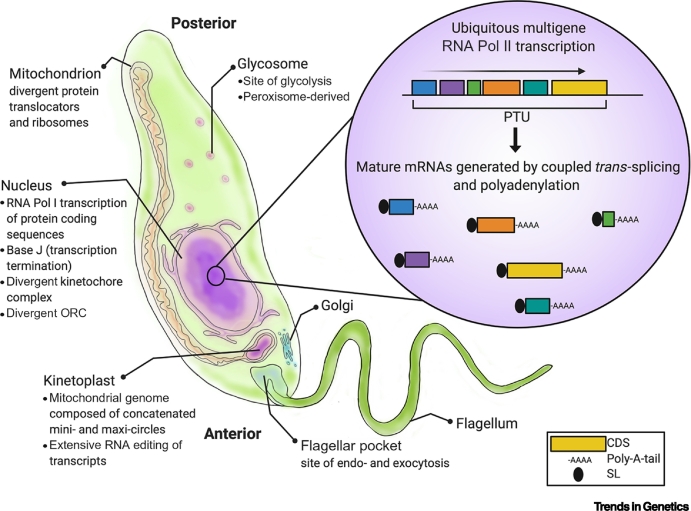
Schematic Structure of a *Leishmania* Promastigote Cell. See Box 1 for details. Abbreviations: CDS, coding DNA sequence; ORC, origin recognition complex; PTU, polycistronic transcription unit; SL, splice leader.

Alt-text: Box 1

Increasing evidence suggests that novelty in kinetoplastid biology is also seen in the machineries needed for nuclear genome transmission. DNA replication is initiated at specific genomic loci called origins. In eukaryotes, origins are defined by the binding of the origin recognition complex (ORC), which pinpoints where the replisome is to be recruited and, thus, where DNA replication begins. Biochemical analysis of ORC composition in *T. brucei* [36,37], allied to homology-based surveys of ORC subunit presence and absence across eukaryotes [10,38,39], indicate kinetoplast ORC subunit number and/or conservation differs from the frequently described, canonical six subunit ORC structure [40,41] (see Box 2 for a fuller discussion). Perhaps even more dramatically, the kinetoplastid kinetochore complex, which connects spindles to centromeres during mitosis, is composed of highly diverged subunits, with those predicted in the structure to be positioned more proximal to the genome defying any identification of sequence homology with kinetochore subunits in other eukaryotes [42,43]. Perhaps, then, the unusual nuclear genomes of kinetoplastids has necessitated innovation in the transmission machineries (Box 2), for reasons that remain unexplained but could be a source of novel therapeutics against parasitic kinetoplastids [44].

Box 2 **The Diverged Origin Recognition Complex of Kinetoplastids**

DNA replication initiation at origins occurs through binding of a six protein origin recognition complex (ORC), which then recruits the replicative MCM (minichromosome maintenance) helicase, via two mediators (Cdc6 and Cdt1), to form the pre-replication (pre-RC) complex prior to S phase. During S phase, the complex is activated (to recruit the replisome) and partially disassembled (to avoid re-replication).

Initial studies suggested *T. brucei* encodes just a single ORC-related protein, which was termed ORC1/CDC6, but later work revealed a multisubunit ORC. However, only four putative ORC subunits have been described in *T. brucei* to date, suggesting kinetoplastids may lack Orc3 and Orc6 subunits. Moreover, whereas relatively well-conserved Orc1- and Orc4-like proteins are present in *T. brucei*, putative Orc2 and Orc5 subunits are notably poorly conserved, suggesting asymmetric structural conservation in the complex.

Why kinetoplastid ORC structure is variant is unclear but may be due to the multigene transcription strategy of kinetoplastids (Box 1). ORC1/CDC6 localises to the start and ends of the polycistronic transcription units (PTUs) in *T. brucei*. Functional interaction between kinetoplastid ORC and RNA polymerase, or specific chromatin at transcription start and stop sites, may have arisen to cause such spatial limitation and may have necessitated restructuring of the complex (Figure I). However, early-replicating centromeres in *T. brucei* are bound by a highly unusual kinetochore, which may also recruit ORC or dictate its activity.

An alternative suggestion for kinetoplastid ORC divergence may reside in pre-RC formation. Each of the six kinetoplastid MCM subunits appear well conserved, suggesting the DNA replication machinery downstream of ORC is more conventional. However, a further *T. brucei* ORC-like factor, termed ORC1B, is expressed only in S phase, meaning it behaves unlike any known eukaryotic ORC subunit or Cdc6. ORC1B interacts with ORC (ORC1/CDC6) and MCM (MCM3) and may then limit pre-RC formation to S phase. Alternatively, given the lack of Cdt1 detection in kinetoplastids to date, ORC–MCM may, like in archaea, interact directly but only be activated in S phase once bound by ORC1B. Whether such putative altered regulation might have necessitated divergence of some ORC subunits (e.g., at the ORC–MCM interaction interface) is unclear, but it is conceivable that ORC and/or pre-RC interact loosely with the genome during G1 in order to limit impeding transcription, and only in S phase is tight interaction with origins induced to initiate DNA replication. However, no work to date has demonstrated *T. brucei* ORC1B truly is an ORC component and therefore neofunctionalization cannot be ruled out.

**Figure I:**
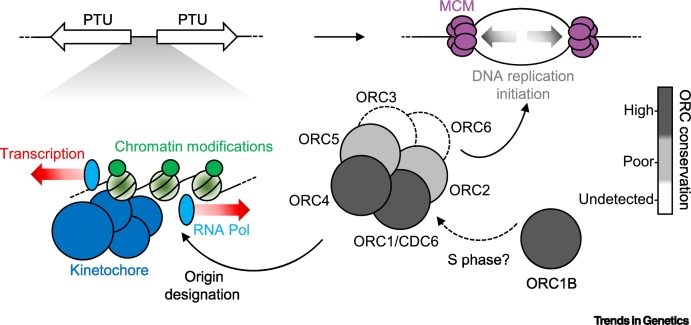
Potential Mechanistic Explanations for Diverged Composition of Kinetoplastid ORC. Abbreviations: MCM, minichromosome maintenance; PTU, polycistronic transcription unit.

Alt-text: Box 2

## DNA Replication Programme of *T. brucei*

To date, genome-wide analysis of DNA replication dynamics in *T. brucei* has been limited to **marker frequency analysis coupled with deep sequencing (MFA-seq)** [45., 46., 47., 48.], a genome sequencing strategy comparing DNA content in replicating and non-replicating cells to identify DNA replication initiation sites and infer replication fork movement (Figure 1A). Another study used **DNA combing** [49], but such experiments have limited capacity to relate DNA replication dynamics to genome organisation. These studies reveal that the programme of DNA replication in *T. brucei* is shaped by genome compartmentalisation and by the ubiquity of multigenic transcription.

Mapping the genomic localisation of one *T. brucei* ORC component, ORC1/CD6, revealed binding only at the boundaries of the PTUs in the genome core, with MFA-seq revealing DNA replication initiation at what appears to be a rather invariant subset of these sites (Figure 1A; see later). In *Schizosaccharomyces pombe* **pre-replication complex**es (**pre-RCs**; Box 2) are excluded from transcribed regions of the genome [50]. Moreover, long genes in eukaryotes more frequently harbour **common fragile sites**, due to transcription overlapping with DNA replication [51,52]. Limitation of ORC to PTU boundaries may be critical in kinetoplastids to prevent the complex from interfering with RNA Pol movement during transcription, as this would be severely detrimental.

Only 20–25% of ORC1/CDC6-binding sites appear to display origin activity, with such sites conserved in different *T. brucei* strains and in two life cycle stages (Figure 1A) [45,46]. What, if anything, distinguishes mapped, active origins from ORC1/CDC6-bound, origin-inactive sites is unclear (with the exception of the single origin per chromosome that overlaps with the mapped centromere). Nonetheless, mapping of only ~50 origins reveals greater interorigin spacing (~400 kb in the ~22 Mb core genome) than described in non-kinetoplastid eukaryotes (Figure 1A), and close to the widest interorigin spacings described in the larger genomes of metazoans [36,46,53]. Indeed, modelling of MFA-seq mapping suggests *T. brucei* operates using close to or below the minimum number of origins needed to complete genome duplication during S phase [54]. Thus, localising ORC and the pre-RC to PTU boundaries may alone be insufficient to limit transcription–replication clashes. What challenges such an origin-poor genome poses for maintenance is unclear, but in other eukaryotes chromosomes that are denuded in, or devoid of origins display increased mutation and instability [53,55]. In fact, even when using a minimum number of origins, clashes between the replisome and RNA Pol are unavoidable and, in kinetoplastids, potentially highly localised. In other eukaryotes such clashes result in damage and accumulation of R loops [56,57] but, to date, mapping in *T. brucei* has not detected signals of these events within the transcription units [30,31]. Thus, we cannot exclude the use of flexible DNA replication initiation events that have so far escaped detection, or uncharacterised mechanisms to efficiently restart stalled DNA replication and transcription.

Less is known about replication of the large, variable *VSG*-rich *T. brucei* subtelomeres (Figure 1B), since their repetitive sequence content limits mapping of short read sequences. Though ORC1/CDC6 appears to bind abundantly in this genome compartment [45], it is unclear if this binding leads to widespread DNA replication initiation or if the protein, with or without ORC, provides another role [58]. In addition, it is unclear if the subtelomeres are late replicating or if their DNA replication timing or pattern might relate to variability. Recent long-read sequencing has substantially improved assembly of the subtelomeres in one *T. brucei* strain [15], but we cannot yet predict what pathways (e.g., mutation and **recombination**) lead to variability during growth, infection, and transmission. Long-read sequencing does, however, reveal the centromeres of the three largest chromosomes to be positioned within the subtelomeres [15]. It will therefore be useful to know if these centromeres, like those in the smaller chromosomes [36], provide early-replicating origins, as this may shed light on determinants of replication timing. Indeed, no work to date has examined the connection between origins and centromeres or has asked if divergence in both ORC and the kinetochore in kinetoplastids might have a basis in functional interdependence (Box 2).

## Targeted DNA Replication to Drive *T. brucei* VSG Switching?

**Figure 2:**
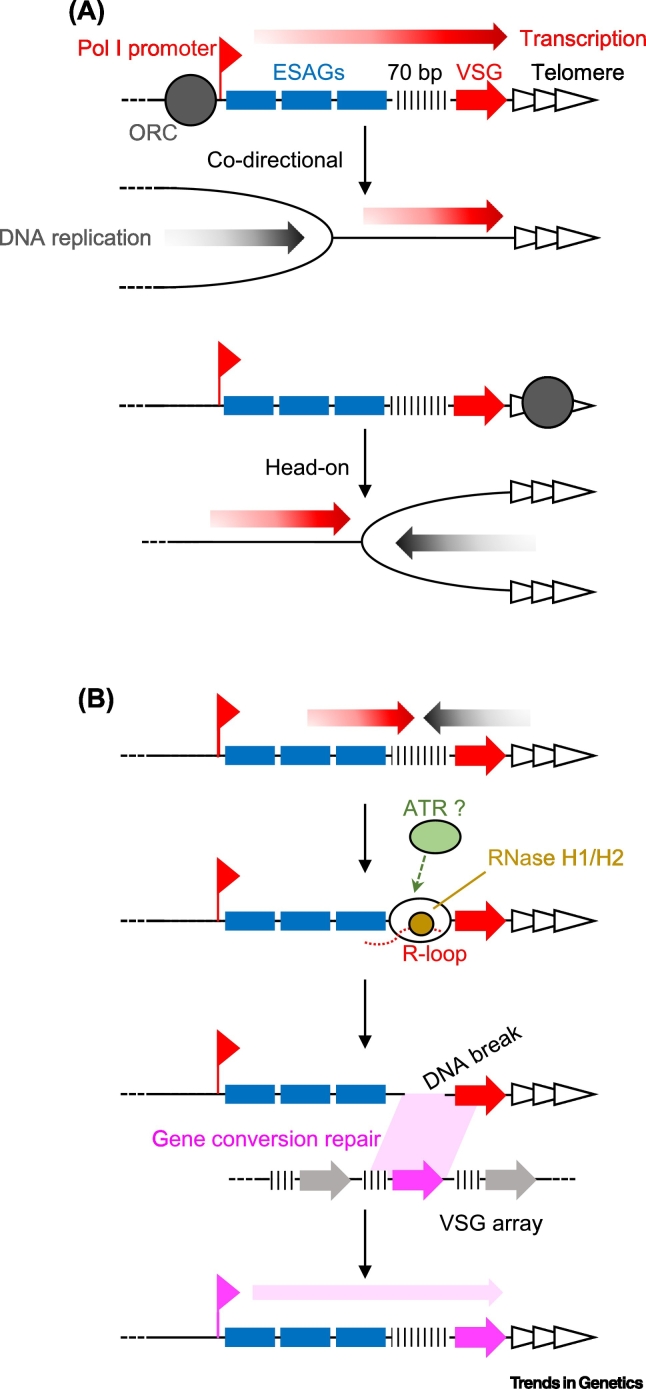
DNA Replication and Transcription Intersect to Drive Antigenic Variation in the *Trypanosoma brucei* VSG Expression Sites. (A) Current understanding of DNA replication in the actively transcribed *T. brucei* VSG ES. A simplified VSG ES is shown, with key features indicated: RNA Pol I promoter, transcription direction (red arrow), ESAGs (blue boxes), 70 bp repeats (hatches), VSG (red arrow), and telomere (array of arrows). Two possibilities for the direction of ORC-derived DNA replication are shown (black arrows), which can result in codirectional or head on collisions with transcription. ORC binding in the ES has not been mapped, however, and so it remains possible that DNA replication is ORC-independent (not shown). (B) A model for VSG recombination during antigenic variation. Transcription is impeded by DNA replication, here shown as a head on collision and focused on the 70 bp repeats. Pausing of RNA Pol I leads to the formation of an RNA–DNA hybrid (R loop), in which RNase H1 and RNase H2 (yellow circle) hydrolyse the RNA to resolve the structure. ATR (green circle) may recognise the R loop. Together, these activities contribute to the generation of DNA breaks in the ES, which are repaired by gene conversion from a silent VSG (pink arrow, here shown in a subtelomeric array) into the ES, replacing the previously expressed VSG. Abbreviations: ATR, Ataxia telangiectasia and Rad3-related; ES, expression site; EASG, ES-associated gene; ORC, origin recognition complex; RNA Pol, RNA polymerase; VSG, variant surface glycoprotein.

By contrast with the relative rigidity of origin activity in the constitutively transcribed *T. brucei* core genome, the loci where *VSG*s are expressed display dynamism in DNA replication activity. *VSG*s are transcribed, by RNA Pol I, from multigene transcription units termed VSG expression sites (ESs), which are found directly adjacent to the telomere (Figure 2). Approximately 15 ESs are present in the genome but only one is actively transcribed at a given time, though the identity of the transcribed ES is not fixed [59]. The timing of ES replication is dictated by its transcription status: whichever ES is actively transcribed, that single locus is replicated early in S phase, whereas all silent ESs are replicated late [46]. Furthermore, all ESs are replicated late in S phase in insect-stage *T. brucei* cells, where transcription of all these loci is silenced [46]. These data reveal that regulated transcription in *T. brucei* can affect DNA replication activity and suggest a basis for understanding targeted *VSG* gene recombination into the ES to cause surface VSG coat switching during antigenic variation (Figure 2).

How active transcription of one ES is linked to early DNA replication is unknown (Figure 2A). The simplest explanation might be that an ORC is recruited to the RNA Pol I promoter in the active ES, leading to conventional bidirectional DNA replication across the ES towards the telomere, and towards the chromosome core. However, ORC localisation has not been mapped to the ESs and distinguishing binding to active and inactive promoters would be complex due to promoter sequence conservation. In addition, regulation of ES transcription may not be due to control at the point of initiation but through limiting transcription elongation to the active ES [59,60]. If so, why an ORC would only be recruited to the promoter of the active ES promoter is unclear. In a number of eukaryotes, ORCs have been shown to associate with the telomere [61., 62., 63., 64., 65.]. In *T. brucei*, ORC1/CDC6 also interacts with telomeres, but without the involvement of **shelterin** [66], and RNAi results in increased transcription from previously silent ESs [45,66]. If such ORC telomere interaction led to pre-RC formation, DNA replication may initiate and proceed towards the ES promoter but, again, why this would occur earlier in the active ES is unexplored. Irrespective, either form of active ES-targeted DNA replication could cause clashes with RNA Pol I transcription, leading to damage and forcing repair by recombination using silent *VSG*s as sequence templates (Figure 2B). Recent evidence is consistent with such a model. First, R loops, which can form at sites of replication–transcription clashes [52,57,67], can be detected across the active ES and become more abundant when their resolution is hampered by loss of RNase H1 [68] or RNase H2 [30], which also leads to increased damage in the ES and increased VSG switching. Second, loss of the damage signalling protein kinase **Ataxia telangiectasia and Rad3-related (ATR)**, which has been implicated in recognising and resolving DNA replication impediments, including via R loops [69], similarly leads to increased ES damage and VSG switching [70]. Targeting of recombination to the active ES, with concomitant formation and resolution of strand exchange intermediates, may explain why the duplicated active ES is late to segregate during mitosis relative to the inactive ESs [71], as well as explaining increased **crossover recombination** in *T. brucei* **RecQ** [46] and **topoisomerase** mutants [72]. However, one complication is that increased VSG switching in RNase H and *ATR* mutants is not simply the result of greater VSG recombination but also because of increased transcriptional activation of silent ESs, where damage also accumulates. In addition, loss of mini-chromosome maintenance-binding protein (MCM-BP) has been shown to impair DNA replication genome-wide and to result in increased transcription from silent ESs [48]. Thus, it has so far proved hard to separate recombination and transcription events when R-loop processing and damage signalling are impaired, and more detailed analysis is needed of DNA replication rate and direction across the ES. In particular, MFA-seq lacks the resolution needed to map DNA replication dynamics across the ~50 kb ESs, and no experiment has tested how and where transcription and replication intersect within the active ES.

## DNA Replication in *Leishmania*: Beyond the Constraints of Convention

MFA-seq analysis in two *Leishmania* species revealed a dramatic difference in DNA replication programme compared with *T. brucei*, since only one putative origin could be detected in each chromosome during S phase (Figure 1A) [73]. Each MFA-seq predicted *Leishmania* origin localises to the end of a PTU (as in *T. brucei*) and, moreover, appears coincident with a putative centromere [74]. Furthermore, leading strand DNA replication and transcription emanating from each origin are codirectional [75], suggesting that, like in *T. brucei*, clashes between the replisome and RNA Pol are minimised [45].

**Figure 3:**
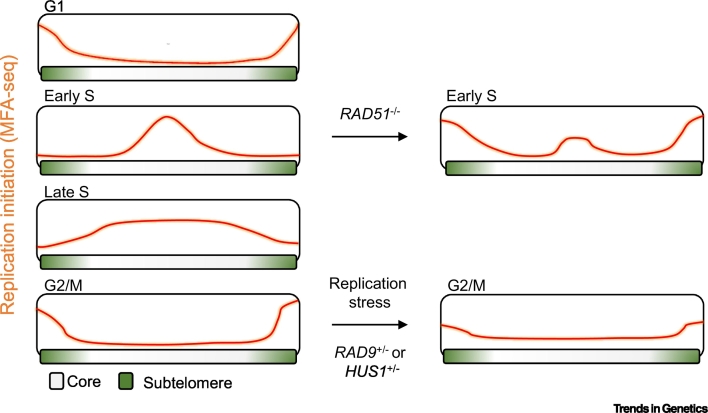
Plasticity of the *Leishmania* DNA Replication Programme. Sites of DNA replication initiation in the different cell cycle phase of *Leishmania*, as detected by MFA-seq analysis, are depicted. Left, a schematic diagram representing DNA replication initiation activity at the indicated cell cycle stages. A hypothetical chromosome is represented in which the core (internal white region) and subtelomere (terminal green regions) are highlighted. Replication (peaks, orange line) of subtelomeres is more obviously detected during G1 and G2/M phases, whereas the core is replicated between early and late S phase. Right, a schematic diagram representing DNA replication initiation activity in distinct conditions. Upon loss of RAD51 DNA replication initiation at chromosome cores during S phase is impaired. This impairment is counterbalanced by increased DNA replication activity at the subtelomeres, which is normally confined to G2/M and G1 phases. Upon replication stress or deficiency of RAD9 or HUS1, DNA replication activity at subtelomeres during G2/M is reduced. Abbreviation: MFA-seq, marker frequency analysis coupled with deep sequencing.

Although the total number of MFA-seq-mapped origins in *Leishmania* and *T. brucei* are not dramatically different, reflecting similar total genome sizes in the parasites, the larger chromosome number (33–36) and wider range of chromosome size (0.25–3.3 Mb) in *Leishmania* predicts that a single origin per chromosome is insufficient to ensure complete genome duplication during S phase [73,76]. Thus, unlike *T. brucei*, *Leishmania* appears to operate with less than the minimum predicted origin number, implying a highly unorthodox DNA replication programme. Expanded MFA-seq of several cell cycle stages provides evidence for such unorthodoxy [75]. First, the length of time to complete a chromosome’s replication is dependent on its size. Second, the DNA replication timing of each chromosome appears temporally compartmentalised, with duplication of the core being confined to S phase, whereas duplication of subtelomeres is detected during late S, G2/M, and G1 phases of the cell cycle (Figure 3). Third, DNA replication of the core and subtelomere compartments seems to rely on distinct machineries, since replication of chromosome subtelomeres, unlike the core, is sensitive to replication stress and depends on at least two DNA replication stress response factors: RAD9 and HUS1 [77., 78., 79.]. Thus, post-S phase DNA synthesis may be an integral feature of *Leishmania*’s DNA replication programme, perhaps to a greater extent than is recognised in other eukaryotes [80,81].

DNA replication outside S phase is frequently seen in cells with aneuploidy [82,83], so a reliance on such activity to ensure genome duplication may underlie the widespread aneuploidy found in *Leishmania* [84,85]. Also, the highly mutagenic nature of DNA synthesis outside S phase [86., 87., 88.] may focus increased mutation rate on the subtelomeres, facilitating adaptive change. In this regard, *Leishmania* subtelomeres are particularly prone to copy number variation [22] and so, despite being less extensive than *T. brucei* subtelomeres, they may also be read–write genomic rearrangement hotspots. Indeed, post-S phase subtelomere replication may be worth exploring in *T. brucei* and wider pathogens whose survival depends upon antigenic variation.

## DNA Replication in *Leishmania*: a Highly Flexible Process?

**Short nascent DNA strand sequencing (SNS-seq)** mapping and DNA combing analyses in asynchronous *Leishmania* populations have made clear that our understanding about the DNA replication programme in this parasite is incomplete. SNS-seq detects >5000 replication initiation sites, the vast majority spread across the PTUs and, hence, spatially distinct from highly localised, MFA-seq-predicted origins [89]. DNA combing has revealed DNA synthesis at >1 site in a single DNA molecule, though without reference to the genome [49,89]. If the events identified by SNS-seq are true origins, then the number predicted reveals origin density far exceeding that found in any other eukaryote [53]. Also, ORC and pre-RC loading onto each SNS-seq site during G1 phase would represent a considerable impediment to RNA Pol passage. By contrast, if each site is used only rarely in a cell, with the SNS-seq mapping representing population diversity, then the data may reveal stochastic DNA replication initiation acting alongside the relatively defined programme detected by MFA-seq. Indeed, we cannot yet say if a related process occurs but has not been detected in *T. brucei*. Lombraña *et al.* [89] suggested that SNS-seq maps an interconnection between transcription and DNA replication initiation in *Leishmania*. While such association is also seen in other eukaryotes [90,91], the predominance of multigene transcription in kinetoplastids may mean the mechanisms linking these reactions are divergent. In fact, SNS-seq may have revealed how kinetoplastids respond to conflicts between the replisome and RNA Pol, with implications for genome stability that have not so far been explored. Correlation of all DNA replication data with genome features such as G4 structures [92] and R loops [31] may be informative.

## Impairment of Homologous Recombination Reveals Plasticity in *Leishmania* DNA Replication

Homologous recombination (HR) factors have been shown to mediate the generation and/or stability of episomes in *Leishmania* [18,19,93., 94., 95., 96.]. Other work has suggested that loss of some HR factors may be lethal in *Leishmania*, suggesting unexplored cellular roles [94,97]. One such role has been revealed by analysis of conditional RAD51 and RAD51-3 mutants in *Leishmania major* [98]. Loss of both HR factors results in impaired S-phase DNA synthesis in the parasite. More strikingly, in the absence of RAD51, initiation of DNA replication around the single MFA-seq predicted S phase origins in each chromosome is decreased, while increased DNA replication is seen around the subtelomeres (Figure 3). Thus, loss of a central HR enzyme alters the DNA replication programme of *Leishmania*. The basis for this change remains to be determined but one possibility is that RAD51 and RAD51-3 play indirect and distinct roles in genome duplication by driving re-initiation of stalled DNA replication along chromosomes, ensuring replication forks emanating from the core reach the subtelomeres. Alternatively, given that *Leishmania* origins might exclusively localise at centromeres, they may be especially vulnerable to breakage during chromosome segregation. RAD51 may then be required to repair such injuries in order to maintain the origins and allow their proper licensing in the next cell cycle. This role may be less important in *T. brucei*, where origins are not constrained to colocalise with centromeres. It is important to note that, despite the conservation of ORC components across kinetoplastids [36,37,99,100], no functional analysis of the complex has been performed in *Leishmania*, including mapping of their binding sites in the genome. Therefore, it remains to be examined whether innovations in ORC composition and structure shape the physical, temporal, and functional compartments of the DNA replication programme in this parasite, as well as their potential cooperation with the HR machinery.

Recombination-directed DNA replication initiation has been described in viruses [101., 102., 103.], bacteria [104], polyploid archaea [105], and *Tetrahymena* [106]. However, such interconnections are not focused on DNA replication origins but, instead, assume importance when a cell’s initiator-driven origin activity is ablated. Nonetheless, co-opting RAD51 to act in both DNA replication and genome variation may be a strategy *Leishmania* has evolved to consolidate genome stability and plasticity. Moreover, the dramatic change in the DNA replication landscape, including increased subtelomere replication, upon RAD51 ablation reveals the remarkable plasticity of *Leishmania*’s DNA replication programme. Such malleability could be a useful adaptation strategy, perhaps with so far undetected parallels for read–write genome alterations in *T. brucei*.

## Do *T. brucei* and *Leishmania* Provide Lessons for Read-Write Adaptation in Other Pathogens?

Many questions remain to be answered about read–write processes and DNA replication programming even within the two kinetoplastid parasites we have discussed (see Outstanding Questions), but parallels may be found elsewhere. For instance, *T. cruzi* also displays genome-wide aneuploidy [107] and ~50% of its genome comprises variable multigene families, with evidence for recombination within them [108,109]. *Plasmodium* parasites not only undergo ploidy variation by schizogony during their life cycle but rely on antigenic variation, which uses subtelomeric gene families whose diversification has been linked to DNA replication [110]. *Trypanosoma congolense* and *Trypanosoma vivax* also rely on antigenic variation, but no equivalent mechanistic analysis has been conducted into the reactions [111]. The emerging data we have described in *T. brucei* and *Leishmania* suggest that an intimate connection between genome transmission and purposefully generated genome changes may be a widespread strategy employed by pathogens to efficiently adapt to changes between hosts and perpetuate infection.

## Concluding Remarks

The advent and continued development of genome sequencing strategies has allowed improved understanding of the dynamics of DNA replication and has revealed connections with read–write genome adaptations in *T. brucei* and *Leishmania*. Further mechanistic dissection of these connections is now needed and may be expanded and improved by whole-genome screens, such as through CRISPR or RNAi. In addition, as many of the processes we have discussed involve multigene families and other repeats that are problematic for short-read DNA sequence analysis, application of long-read sequencing approaches will be illuminating, as will exploring how the replication and repair reactions relate to nuclear and genome ultrastructure. Addressing these questions in a wider range of pathogens, and in their free-living relatives, will reveal the purposes and evolution of these reactions, which may then provide strategies to develop new therapies against neglected parasites.

Outstanding Questions How do kinetoplastids manage and potentially utilise clashes between DNA replication and transcription in the polycistronic transcription units?

Where does ORC bind in the *Leishmania* genome and what is the connection, if any, between ORC localisation and origin activation at transcription initiation/termination sites and centromeres in kinetoplastids?

How do the divergencies in ORC composition and structure correlate with the peculiarities of DNA replication programmes in kinetoplastids?

Are there ORC-independent activities that provide more flexible or less localised DNA replication initiation in kinetoplastids?

Have all sites of DNA replication initiation been detected by MFA-seq analysis of *T. brucei*?

How are the transcriptionally silent *T. brucei* subtelomeres replicated?

What is the nature of early DNA replication of the actively transcribed ES in *T. brucei*: is this ORC-dependent; what is the direction, rate, and the influence of DNA sequence and chromatin structure?

In our model for DNA replication-directed VSG switching, is only HR repair induced or are other repair reactions, such as microhomology-mediated end joining (MMEJ), elicited?

What is the nature of replication initiation activity detected by SNS-seq in *Leishmania*? Can this be also be detected in other kinetoplastids?

What is the mechanism by which homologous recombination contributes to DNA replication in *Leishmania*?

What machinery and epigenetic features directs subtelomeric DNA replication in *Leishmania*, and how accurate is the reaction?

Beyond the two *T. brucei* stages studied to date, how does the DNA replication programme vary between life cycle stages of kinetoplastids?

Alt-text: Outstanding Questions

## Glossary

**Aneuploidy**: altered ploidy of one or more chromosomes. In most contexts, aneuploidy has detrimental effects on cellular physiology and is frequently seen in cancer. By contrast, aneuploidy is a constitutive feature of *Leishmania*’s genetic makeup.
**Antigenic variation**: the process by which a pathogen changes an antigen expressed on the cell surface in order to evade host adaptive immunity. Mechanisms of antigen switching vary between pathogens. In *T. brucei*, antigenic variation involves switching of VSG, of which ~10^7^ copies of a single type are expressed in a single cell at one time generating a protective coat. Recombination and transcription control are both used to allow *T. brucei* VSG switching.
**Ataxia telangiectasia and Rad3-related (ATR)**: member of the PI3 kinase-like family of protein kinases (PIKKs). ATR is one of a number of protein kinases involved in the sensing and resolution of DNA damage, with a focus on DNA replication impediments.
**Common fragile site**: region within a chromosome that is prone to breakage under replicative stress pressure.
**Crossover recombination**: one possible recombination outcome during repair of double-stranded DNA breaks by homologous recombination. When a Holliday junction (a four-stranded branched recombination intermediate) is resolved, a crossover can occur where a DNA stretch from one side of the break is swapped over with the corresponding DNA stretch in the homologous template.
**DNA combing**: a method routinely used for studying DNA replication dynamics. It relies on labelling newly synthesized DNA *in vivo* using labelled thymidine analogues. Following isolation and uniform stretching on a glass surface, sites of labelled nucleotide incorporation in individual DNA molecules are visualised by immunostaining.
**DNA repair**: a collection of pathways involved in the repair of DNA lesions. Initiated by DNA damage sensing, including by DNA-repair-dedicated protein kinases such as ATR, which then enact the appropriate pathway. Examples of repair pathways are nucleotide excision repair, which corrects bulky DNA lesions; base excision repair, which corrects DNA base damage; mismatch repair, which corrects aberrant base pairing; and homologous recombination, nonhomologous end-joining and microhomology-mediated end-joining, which correct DNA breaks.
**DNA replication**: a semiconservative reaction in which parental double-stranded DNA is duplicated prior to cell division.
**Episome**: an extrachromosomal DNA element that is capable of being transmitted through cell divisions independently from the cell’s genome.
**Marker frequency analysis coupled with deep sequencing (MFA-seq)**: a method used to infer DNA replication initiation, progression, and timing. It relies on the assumption that during S phase, DNA copy number increases around sites of initiation (origins) as replication progresses. Therefore, the depth of short-read sequence mapping in replicating cells (S phase) is normalised relative to non-replicating cells (G1, G2, or S phase), revealing genomic loci where there is ongoing replication.
**Ploidy**: the number of homologous chromosomes in a given cell and organism.
**Pre-replication complex (pre-RC)**: a protein complex that forms on replication origins prior to S phase and is composed of the ORC, Cdc6, Cdt1 and the MCM heterohexamer (see Box 2 for more details).
**R loops**: a three-stranded nucleic acid structure involving a DNA–RNA hybrid and displaced single-stranded DNA.
**Recombination**: process by which DNA sequence content is altered, frequently during DNA breaks repair. The reaction can be catalysed using an unbroken, homologous DNA molecule as template for break repair, or may involve re-ligation of the broken molecules without a template.
**RecQ**: family of DNA helicases with roles in multiple genome processes such as DNA replication, recombination, and repair.
**RNase H**: a family of endonuclease enzymes that catalyse the cleavage of the RNA moiety in an RNA–DNA hybrid.
**Shelterin**: a complex of proteins (in mammals: TRF1, TRF2, RAP1, TIN2, TPP1, and POT1) that associate with the telomeric repeats and adjacent sequences at chromosome ends.
**Short nascent DNA strand sequencing (SNS-seq)**: a method used for genome-wide mapping of origins of replication that relies on selective enzymatic digestion of long DNA strands (without a RNA primer) to enrich for short newly synthesized DNA strands (containing an RNA primer), which are present around recently fired origins of replication.
**Topoisomerase**: a class of enzymes that introduces nicks or breaks into the DNA backbone, followed by resealing; can be used, for instance, to alleviate overwinding of the DNA helix ahead of a replication fork.
